# Development and pilot testing of a patient‐reported outcome measure to assess symptoms of parastomal hernia

**DOI:** 10.1111/codi.16850

**Published:** 2024-01-04

**Authors:** Jane M. Blazeby, Charlotte Murkin, Leila Rooshenas, Daisy Elliott, Kerry Avery, Katy Chalmers, Sian Cousins, Thomas Pinkney, Natalie Blencowe, Barnaby C. Reeves, Neil Smart

**Affiliations:** ^1^ Population Health Sciences and Bristol Biomedical Research Centre University of Bristol and University Hospitals Bristol and Weston Foundation Trust Bristol UK; ^2^ Birmingham University Hospitals NHS Trust Birmingham UK; ^3^ Royal Devon and Exeter Hospital Exeter UK

**Keywords:** cognitive interviews, parastomal hernia, patient reported outcomes, pilot testing, questionnaire development

## Abstract

**Aim:**

The aim was to develop and pilot a patient‐reported outcome measure (PROM) to assess symptoms of parastomal hernia (PSH).

**Methods:**

Standard questionnaire development was undertaken (phases 1–3). An initial list of questionnaire domains was identified from validated colorectal cancer PROMs and from semi‐structured interviews with patients with a PSH and health professionals (phase 1). Domains were operationalized into items in a provisional questionnaire, and ‘think‐aloud’ patient interviews explored face validity and acceptability (phase 2). The updated questionnaire was piloted in patients with a stoma who had undergone colorectal surgery and had a computed tomography scan available for review. Patient‐reported symptoms were examined in relation to PSH (phase 3). Three sources determined PSH presence: (i) data about PSH presence recorded in hospital notes, (ii) independent expert review of the computed tomography scan and (iii) patient report of being informed of a PSH by a health professional.

**Results:**

For phase 1, 169 and 127 domains were identified from 70 PROMs and 29 interviews respectively. In phase 2, 14 domains specific to PSH were identified and operationalized into questionnaire items. Think‐aloud interviews led to three minor modifications. In phase 3, 44 completed questionnaires were obtained. Missing data were few: 5/660 items. PSH symptom scores associated with PSH presence varied between different data sources. The scale with the most consistent differences between PSH presence and absence and all data sources was the stoma appearance scale.

**Conclusion:**

A PROM to examine the symptoms of PSH has been developed from the literature and views of key informants. Although preliminary testing shows it to be understandable and acceptable it is uncertain if it is sensitive to PSH‐specific symptoms and further psychometric testing is needed.

## INTRODUCTION

Formation of a stoma (e.g., colostomy) is often necessary following bowel resection. Like other surgical interventions it can be associated with complications. One complication is the development of a parastomal hernia (PSH), defined as an ‘abnormal protrusion of the contents of the abdominal cavity through the abdominal wall defect created during placement of stoma’ [1, 2, 3, 4, 5]. Estimates of the rates of PSH development range from 30% by 12 months to over 50% in the longer term [1, 2, 3, 4, 5, 6, 7, 8]. Some PSHs are small and asymptomatic; others lead to serious problems. Bowel obstruction and strangulation can occur; this may require emergency surgery. Patients may experience psychological problems associated with concerns over large abdominal bulges and/or anxiety related to stoma appliance leakage around the PSH. Prevention and detection of PSH is therefore important [1, 2, 3, 4, 5, 6, 7, 8].

The CIPHER study was funded by the UK National Institute of Health Research to examine these issues [9, 10, 11]. CIPHER has two phases. Phase A aims to create methods for recording technical risk factors of PSH at the time of stoma formation [12] and to develop and pilot a patient‐reported outcome measure (PROM) for capturing PSH symptoms, although it is uncertain that this will be possible. Using symptoms alone to diagnose PSH is notoriously unreliable [7]. Some patients describe stoma pain and bulging but the same symptoms may occur in patients with a stoma without evidence of PSH (related to scar pain, the stoma appliance or peristomal skin problems). If a specific PSH PROM could be developed this could be valuable for monitoring patients with a stoma during follow‐up. There has been increased use of abdominal computed tomography (CT) scan to diagnose PSH, but interpretation can be challenging and systems for PSH classification have poor evidence of validation [7]. Phase B aims to utilize the outputs of phase A in a cohort study of patients undergoing stoma formation to examine PSH incidence, symptoms and health economic impact [9, 10, 11]. In phase B the cohort data will be used to undertake psychometric testing of the PROM for PSH symptom assessment developed in phase A. This paper presents CIPHER phase A work and describes the development and pilot testing of a PROM for assessment of symptoms of PSHs.

## METHODS

Standard phased questionnaire development methods were used [13]. Here, phases 1–3 are reported. The final part of the development (phase 4) consists of psychometric testing which is planned as part of phase B of the main CIPHER study, and it will be reported elsewhere.

### Phase 1: Literature analyses and interviews to create a list of potential domains relating to symptoms of PSH

Literature searches were initially aimed to identify a PSH‐specific PROM. None was identified. It was therefore decided to examine PROMs developed for patients with colorectal cancer [14]. All scales and items were extracted and examined by two independent researchers to identify domains associated with PSH. The lists were compared and a final list agreed. The domains informed the interview topic guides.

Semi‐structured interviews were undertaken with patients and health professionals to explore perspectives and experiences relating to symptoms associated with PSH. Participants were recruited for practical reasons from two National Health Service trusts in England. Eligible patients included individuals who had undergone colorectal surgery and had a stoma formed. They were identified by the clinical teams and given written information sheets and a consent form. The main researcher (CM) contacted patients who had agreed to participate and arranged an appointment for the interview. Before this started written consent was obtained. Patient participant interviews were conducted in the participant's home or in the hospital depending on the participant's preference. They were audio‐recorded. A purposeful sampling strategy ensured that perspectives were captured from a range of participants, with the intention of building a sample that varied based on age, gender, ethnicity, type of stoma and known PSH problems.

Eligible health professionals included specialist colorectal and general surgeons, and stoma and clinical nurse specialists. Health professionals were identified by JMB and sampled in relation to clinical experience, seniority, health discipline and gender. They were approached by email, with an information sheet, and asked to participate. Interested professionals were contacted by CM who gained written consent prior to the interview. Health professional participants were interviewed in their place of work (e.g., hospital or university office).

Patient and health professional interviews were guided by topic guides to ensure that discussions covered the same core issues but with sufficient flexibility to allow new issues of importance to the participants to emerge. All interviews were audio‐recorded and transcribed verbatim. Transcripts were imported and managed into NVivo (version 10), which was used to facilitate analysis.

The process incorporated guidance for eliciting health domains using qualitative methods [15, 16] relating to symptoms of PSH.

### Phase 2: Interview data analyses and provisional questionnaire development

Data from phase 1 was used to create domains and then operationalized into items to create a provisional PROM. All data relating to PSH were assigned labels (coded) by CM and a more experienced qualitative researcher (KC). Data were analysed using techniques of constant comparison derived from grounded theory methodology, and emerging codes across the dataset were compared to look for shared or disparate views among participants [15, 16]. Data collection and analysis continued until the team was confident that saturation had been reached (i.e., when no more patterns or themes emerged from the data) in relation to the research objective. The domains that arose from the literature analyses were compared and mapped onto the domains arising from patient and professional interviews by CM. The study team reviewed the domains and agreed a final list of domains (which was deliberately inclusive). This was operationalized with three experts into questionnaire items (KA, JMB and BR) with a 4‐point ordinal response scale (except for item 5b about seeking help from a health professional, with simple Y/N response options): not at all; a little; quite a bit; a lot. The provisional questionnaire was organized by overarching themes (hypothesized questionnaire scales) and examined for face validity and acceptability by undertaking interviews using ‘think‐aloud’ techniques [17]. Participants were identified by the clinical teams and CM approached them for consent. The sampling strategy was like that described above. The interviews were conducted at a place of the participant's choice (their home or in the hospital) and consent was gained at the time of the interview. The participant was asked to complete the questionnaire and simultaneously express their thoughts and feelings about the meaning of the items and other aspects of the questionnaire whilst being observed by a researcher. The ‘think‐aloud’ sessions were audio‐recorded, transcribed verbatim and analysed thematically. Items that were noted to be confusing were discussed, reworded for clarity and iteratively examined for acceptability and face validity. Interviews were conducted until no further changes were suggested by interviewees.

### Phase 3: Piloting the questionnaire

The PROM was piloted in three hospital trusts in England where surgeons participating in the study were based (two the same as in phase 1). Eligible were patients with a stoma who had an abdominal CT scan within 12 months. These were identified by clinical staff. The research team distributed postal invites and information about the study including a consent form and a questionnaire to complete and return in a stamped addressed envelope. Sociodemographic age, gender, ethnicity, employment and domestic status data were patient reported and clinical data (date and type of surgery, type of stoma) were collected by CM from the hospital records.

### Presence of a PSH

Data were collected about PSHs from three sources: (i) examination of hospital records by a surgical trainee who had not met the patient (PSH present, not present); (ii) review of the CT scans of participating patients (with CT conducted during routine follow‐up within 12 months of completing the questionnaire) by an experienced colorectal surgeon (NS) (PSH present, not present); and (iii) from participants, in response to the question ‘have you ever been told that you have a PSH?’ (yes, no, unsure).

### Patient‐reported outcome measure and PSH presence data analyses

Questionnaire responses (1, 2, 3, 4) were linearly transformed to a 0–100 scale (0, 33, 66, 100 with a high score meaning more problems) as per questionnaires commonly used in oncology [13]. Scores were summarized by overarching themes (potential scales) and a total questionnaire score, described by group (with or without PSH). Summary scores for the three methods of PSH data source (i)−(iii) are presented alone because of the exploratory nature of the study and the small sample.

## RESULTS

### Phase 1

Of the 70 PROMs identified for patients with colorectal cancer 169 individual issues covering relevant symptoms and problems associated with PSH were extracted. Twenty‐seven interviews (seven surgeons, three stoma nurses, nine patients with PSH and eight with stomata alone) were conducted and from these 127 relevant PSH issues were extracted. Mapping the list of PSH issues from the literature with data from the interviews created 14 PSH‐specific domains: stoma pain, abdominal pain, nausea, problems with stoma appliance fitting, accessing stoma care, skin problems around the stoma, sleep, stoma output, body image, stoma bulging/appearance, leakage from stoma/odour, social impact of stoma, emotional impact of stoma and role/work function. Domains were operationalized into items that formed the provisional questionnaire.

### Phase 2

Think aloud interviews with 16 patients living with and without PSH showed that the questionnaire was acceptable to patients with no complaints about it being offensive or difficult to understand. Minor amendments of the content and layout of the questionnaire (e.g., response categories for one item changed and improvements to the layout of items that required bypassing if a filter question was answered ‘not at all’) were made in response to comments from three patients.

It was agreed by the study team to start the questionnaire with an item asking if a health professional had *ever* told the participant that they had a PSH (response: yes/no/unsure). This was followed by items assessing symptoms and experiences of PSH. Items were categorized into the five potential scales (overarching domains): the appearance of the stoma (two items); pain and discomfort associated with the stoma (three items); seeking help (two items); stomach problems (two items); problems related with using a stoma appliance (six items, physical and psychosocial). Two items were dependent upon the presence of specific symptoms: (i) a bulge around the stoma and problems with body image; (ii) stoma pain and the need for pain relief. At the end of the questionnaire there was the opportunity for participants to comment in free text, if desired, about additional issues they were experiencing in relation to their stomata or PSH/bulge.

### Phase 3

Questionnaires were posted to 109 eligible participants, of whom 44 returned valid consent forms and a completed questionnaire (postal response rate 40%). The only reasons given for declining (*n* = 9) related to ill health or disease progression. The item completion rates were excellent (five single item responses missing in total, four by one participant, across 660 individual items). Patients had a mean age over 60 years (mean 64, range 22–83), were predominantly Caucasian, living with a partner and retired. Twenty‐six had stomata formed following urgent surgery for colorectal cancer, 29 and 15 had colostomies and ileostomies and 21 were men.

#### Parastomal hernia presence

There was little agreement for the presence of a PSH between the hospital records, expert review of CT scan and patient reports of being informed by a health professional that they had a PSH (Table 1). Hospital records recorded fewer PSHs than CT scans or patients' self‐report. The ‘stoma appearance’ scale shows the most consistent and widest range of scores (more problems in patients with a PSH than without) irrespective of source data. Other scales were less discriminatory and showed some conflicting results between patients classified with and without a PSH by the different data sources. The provisional PSH questionnaire is shown in Table 2 by item and scale structure.

**TABLE 1:** Mean questionnaire scale scores by the presence of PSH (using three sources of data).

Source of data	Stoma appearance	Stoma pain	Seek help	Stomach problems	Stoma bag problems
Hospital notes					
PSH yes *n* = 12	70.8	42.4^b^	36.1	40.3	31.0
PSH no *n* = 31^a^	33.9	30.4^c^	23.1	14.5	25.8
Expert CT review					
PSH yes *n* = 23	55.8	34.8	23.9	21.7	25.4
PSH no *n* = 21	34.1	34.5^d^	31.0	22.2	29.4
Patient report					
PSH yes/unsure *n* = 22	64.4	40.9	40.2	33.3	26.8
PSH no *n* = 22	26.5	31.3^e^	14.4	10.6	27.8

*Note*: A high score indicates more problems.

Abbreviations: CT, computed tomography; PSH, parastomal hernia.

^a^ One set of notes not reviewed.

^b^ Based on 11 responses (one missing).

^c^ Based on 30 responses (one missing).

^d^ Based on 19 responses (two missing).

^e^ Based on 20 responses (two missing).

**TABLE 2:** The provisional parastomal hernia (PSH) questionnaire (individual items and scale structure).

Presence of PSH
1. Have you been told by a doctor or a nurse that you have a parastomal hernia?
Stoma appearance scale (mean score items 2a and 2b)
2a. Is there a bulge or lump of any size, around or under your stoma?
2b. Has the bulge/lump affected your overall satisfaction with your body's appearance (body image)?
Pain or discomfort scale (mean score items 3, 4 and 5a)
3. Have you experienced irritation of the skin around your stoma?
4. Have you experienced discomfort due to problems with the stoma or bulge around the stoma?
5a. Have you experienced pain due to problems with the stoma or bulge around the stoma?
Seeking medical help scale
5b. Has the pain required you to seek help from a healthcare professional (e.g., stoma care nurse, doctor, emergency services)?
Mean score (items 5b and 13)
Stomach problems scale (mean score items 6 and 7)
6. Have you felt nauseated or vomited due to a problem with the stoma or bulge around your stoma?
7. Have you experienced the sensation of feeling bloated due to a problem with the stoma or bulge around your stoma (distension)?
Stoma bag/appliance problems (mean score items 8, 9, 10, 11, 12 and 14)
8. Have you had difficulty fitting the stoma bag (stoma appliance)?
9. Have you had problems with keeping the stoma bag (stoma appliance) secure to the skin?
10. Have you experienced leaks of fluid or faeces from the stoma bag (stoma appliance)?
11. Have concerns over leaks or bag fitting stopped you from doing what you want do? e.g., being out of the home, being social or active?
12. Have you had your stoma bag (stoma appliance) modified or swapped with a different appliance because of leaks or difficulty attaching the bag?
13. Have you required help from a healthcare professional (e.g., stoma care nurse, doctor, emergency services) due to difficulty managing your stoma bag (stoma appliance)?
14. Have you been worried by bad smells (odour) from your stoma, other than when emptying the bag?
Total score (mean of all scales [excluding item 1])

*Note*: All item response categories range from ‘not at all’ to ‘very much’ except for item 1 (which requires a ‘yes’, ‘no’, or ‘unsure’ response).

## DISCUSSION

Patients with PSH experience symptoms and problems that impact on quality of life. Assessment of this can be performed with well‐developed and validated PROMs. It was not possible at the start of this study to identify a well‐developed and validated PROM relevant to PSH so this was the focus of this work. Initially domains relevant to PSH were generated from analyses of existing bowel cancer and stoma questionnaires supplemented with views and experiences of patients and health professionals obtained from semi‐structured interviews. Relevant domains were operationalized into items and a questionnaire. This was examined for face validity and acceptability in cognitive interviews with patients. Some pilot testing was undertaken, and scores were examined by PSH presence which was defined in three ways. Whilst the new tool was found to be acceptable to patients it was not possible to determine whether presence of a PSH was associated with specific symptoms or problems in patients with and without PSH (however that was determined). One scale about stoma appearance appeared promising being able to detect differences in PSH presence or absence whatever the source of PSH identity. Further work is ongoing in the CIPHER study phase B to examine the psychometric properties of this new questionnaire in relation to CT analyses and generic measures of quality of life.

This work was novel when initiated. Literature analyses and updated scoping work is still unable to identify a comprehensively developed and validated PROM that is specific for assessing problems associated with PSH, although it is possible that one does exist. Most studies use generic or colorectal‐cancer‐specific measures to examine problems experienced by patients with PSH or they rely on observer‐recorded symptoms or clinical investigations alone [18, 19]. The benefit of a PROM developed with patient input is that it will contain issues of importance to patients and it will be understandable and valid for self‐report. Currently there are few prospective studies that have used validated PROMs in PSH although there is an extensive literature about PROMs and stomata [20]. Twenty‐one PROMs for assessing quality of life in patients with stomata were identified with only one demonstrating content validity, construct validity, reliability and responsiveness assessed [21]. This PROM focused on problems associated with leakage from the stoma and did not include items about pain or bulging. One study examined the association between PSH (assessed with CT scan according to the European Hernia Society classification) after ileal conduit urinary diversion with a PROM that was study specific. The authors defined ‘symptomatic’ PSH according to the presence of hernia‐related discomfort, appliance problems and/or bowel complications. Few PSHs were strictly found to be asymptomatic although little information about the PROM development and validation is included. It is also unclear whether the PROM data were from the patients themselves [22]. Therefore, a PROM specific for PSH that could be used to supplement routine imaging during follow‐up could act as a screening tool to prompt further investigation if PSH‐specific symptoms are identified. A PROM could also be a useful tool in clinical trials aimed at reducing rates of PSH using different surgical stoma formation techniques. Although more work is needed with this preliminary PROM it is an important step in questionnaire development.

The scale assessing symptoms and problems associated with bulging around a stoma seemed to differentiate between patients with and without a PSH irrespective of how that was determined. It is possible that these are the only PSH‐specific patient problems as the others identified in interviews and the literature could plausibly all be related to the stoma alone (e.g., pain, stomach problems and issues related to the appliance). The development of PSH and bulging can be very significant and many patients decide to live with this rather than undergo further surgery and its associated risks. This has been identified by other groups, and measures specifically focusing on body image assessment may be more pertinent to use in assessing PSH‐specific problems [19, 23]. A PROM is unlikely to ever be sensitive enough to diagnose a small PSH (and there are well known challenges with making a diagnosis clinically or radiologically with debate around definitions and terminology [3, 7]). Indeed, a prospective study of quality of life in adults with an ostomy using a validated PROM showed that the occurrence of PSH was not associated with a reduction in quality of life which may indicate that the tool used was not specific or sensitive enough to PSH quality of life issues [24].

A PROM could be a valuable screening tool for patients in follow‐up to identify those needing a clinical examination or further investigation.

The study has limitations that restrain its generalizability. The research was carried out in hospitals in England. Further work to examine the validity and acceptability of the tool for use in other languages and more diverse groups is needed. The study population included only patients with colorectal cancer because of the availability of CT. There is a need to examine the tool in other groups of patients with stomata formed for different reasons. The pilot testing was very limited by the small number of respondents to a postal questionnaire (and we only had ethical approval to send out one invite to complete the questionnaire). A larger study with more patients is required to examine validity and reliability. It will be important to establish if the PROM is able to distinguish between patients who do and do not have a PSH. Although fewer than half of patients invited to take part did so, this is not unexpected when approaching a population of patients with unsolicited postal information alone [25]. Direct approaches by clinical teams are likely to improve response rates and using electronic methods to collect outpatient data is now recommended. A further weakness of this work is that the patients did not undergo a contemporaneous clinical examination. Data were collected from hospital notes alone. It is recommended that future validation of the PSH PROM questionnaire includes contemporaneous imaging, and clinical examination by an expert.

In summary, this paper reports development and early pilot testing of a new PROM to assess PROMs specifically relevant to patients with a PSH. The PROM is short, acceptable to patients and has preliminary data to support its validity. Further work within the CIPHER study and beyond is now needed to complete its validation.

## AUTHOR CONTRIBUTIONS

**Jane M. Blazeby:** Conceptualization; methodology; data curation; supervision; funding acquisition; writing – original draft; writing – review and editing. **Charlotte Murkin:** Data curation; writing – review and editing. **Leila Rooshenas:** Methodology; supervision; writing – review and editing. **Daisy Elliott:** Methodology; supervision; writing – review and editing. **Kerry Avery:** Methodology; supervision. **Katy Chalmers:** Data curation; formal analysis; writing – review and editing. **Sian Cousins:** Methodology; supervision; writing – review and editing. **Thomas Pinkney:** Conceptualization; methodology; writing – review and editing; funding acquisition. **Natalie Blencowe:** Methodology; supervision; writing – review and editing. **Barnaby C. Reeves:** Conceptualization; methodology; funding acquisition. **Neil Smart:** Funding acquisition; writing – review and editing.

## FUNDING INFORMATION

This work was funded by the MRC ConDuCT‐II Hub for Trials Methodology Research, and the NIHR Health Technology Assessment CIPHER grant (Cohort study to Investigate the prevention of Parastomal HERnia, 14/166/01). It was also supported by the Bristol Surgical Trials Centre, the NIHR Bristol Biomedical Research Centre and the Bristol Royal College of Surgeons Surgical Trials Centre.

## CONFLICT OF INTEREST STATEMENT

The authors declare no conflicts of interest.

## ETHICS STATEMENT

National Health Service (NHS) Research and Ethics Committee approval from the East Midlands−Nottingham 1 Research Ethics Committee [16/EM/0155] gained on 1 June 2016. Ethical and local agreements were in place for the NHS trusts prior to commencing the research.

## DECLARATION

The views expressed are those of the author(s) and not necessarily those of the NIHR or the Department of Health and Social Care. Jane M. Blazeby is an NIHR Senior Investigator. NSB is a Medical Research Council Clinician Scientist.

## PATIENT CONSENT STATEMENT

Participating patients provided written informed consent.

## CLINICAL TRIAL REGISTRATION

The main CIPHER study has ISRCTN registration, identifier ISRCTN17573805.

## Data Availability

Data is available on request to the corresponding author.
