# Correction to ‘Development and pilot testing of a patient reported outcome measure to assess symptoms of parastomal hernia’

**DOI:** 10.1111/codi.17161

**Published:** 2024-09-09

Blazeby JM, Murkin C, Rooshenas L, Elliott D, Avery K, Chalmers K et al. Development and pilot testing of a patient‐reported outcome measure to assess symptoms of parastomal hernia. *Colorectal Dis*. 2024;26:364–70. https://doi.org/10.1111/codi.16850

The funding statement for this article was missing. Funding information (Medical Research Council grants G0800800 and MR/K025643/1).

We apologize for this error.

